# Percutaneous electrical nerve field stimulation improves comorbidities in children with cyclic vomiting syndrome

**DOI:** 10.3389/fpain.2023.1203541

**Published:** 2023-06-14

**Authors:** Katja Karrento, Liyun Zhang, William Conley, Zeeshan Qazi, Thangam Venkatesan, Pippa Simpson, B U.K. Li

**Affiliations:** ^1^ Division of Pediatric Gastroenterology, Hepatology and Nutrition, Department of Pediatrics, Medical College of Wisconsin, Milwaukee, WI, United States; ^2^ Division of Quantitative Health Sciences, Department of Pediatrics, Medical College of Wisconsin, Milwaukee, WI, United States; ^3^ Division of Gastroenterology, The Ohio State University, Columbus, OH, United States

**Keywords:** cyclic vomiting syndrome (CVS), abdominal migraine, disorders of gut–brain interaction (DGBI), functional gastrointestinal disorders, auricular neurostimulation, vagus nerve stimulation, percutaneous electrical nerve field stimulation (PENFS)

## Abstract

**Introduction:**

Children with cyclic vomiting syndrome (CVS) frequently suffer from disabling abdominal pain and comorbidities that impair quality of life. A noninvasive, auricular percutaneous electrical nerve field stimulation (PENFS) device is shown to be effective for abdominal pain in children with disorders of gut–brain interaction. We aimed to determine the effects of PENFS on pain, common comorbidities, and quality of life in pediatric CVS.

**Methods:**

Children aged 8–18 years with drug-refractory CVS were enrolled in a prospective, open-label study receiving 6 consecutive weeks of PENFS. Subjects completed the following surveys at baseline, during/after therapy (week 6), and at extended follow-up approximately 4–6 months later: Abdominal Pain Index (API), State-Trait Anxiety Inventory for Children (STAI-C), Pittsburgh Sleep Quality Index (PSQI), and Patient Reported Outcome Measurement Information System (PROMIS) Pediatric Profile-37.

**Results:**

Thirty subjects were included. Median (interquartile range, IQR) age was 10.5 (8.5–15.5) years; 60% were female. Median API scores decreased from baseline to week 6 (*p* = 0.003) and to extended follow-up (*p* < 0.0001). State anxiety scores decreased from baseline to week 6 (*p* < 0.0001) and to extended follow-up (*p* < 0.0001). There were short-term improvements in sleep at 6 weeks (*p* = 0.031) but not at extended follow-up (*p* = 0.22). Quality of life measures of physical function, anxiety, fatigue, and pain interference improved short-term, while there were long-term benefits for anxiety. No serious side effects were reported.

**Conclusions:**

This is the first study to demonstrate the efficacy of auricular neurostimulation using PENFS for pain and several disabling comorbidities in pediatric CVS. PENFS improves anxiety, sleep, and several aspects of quality of life with long-term benefits for anxiety.

**Clinical trial registration**: ClinicalTrials.gov, identifier NCT03434652.

## Introduction

Cyclic vomiting syndrome (CVS) is a disorders of gut-brain interaction (DGBI) that manifests by recurrent episodes of relentless nausea and vomiting (1). The illness is often disabling due to difficulties managing the relentless nausea, vomiting, and abdominal pain during attacks. Quality of life is in fact worse than in children with other DGBI (2). The impact on mental health in children with CVS is considerable and needs better management. Attacks are most commonly precipitated by emotional arousal (3, 4). Comorbidities of anxiety, depression, and sleep disturbances are highly prevalent and underlie the poor quality of life (5, 6). One study documented that comorbid anxiety rather than vomiting severity predicted reduced quality of life (6). Over time, recurrent episodes and failing drug trials result in progressive anxiety and depression. There is thus a need to better address the mental health comorbidities in pediatric CVS with more targeted therapies.

Despite young patient age, limited scientific evidence, and lack of regulatory approval, there is a high rate of antidepressant use in children with CVS (7). These children are at risk of polypharmacy and serious side effects including suicidal ideation (8). There is an urgent and unmet need for safer, non-pharmacological therapies. Percutaneous electrical nerve field stimulation (PENFS) has emerged as a promising, noninvasive auricular neurostimulation therapy with demonstrated efficacy and regulatory approval in children with pain-associated DGBI (9, 10). A recent study of adolescents with DGBI found positive effects on several non-gastrointestinal comorbidities including sleep and anxiety (11). A low vagal tone was documented to predict PENFS response, suggesting that poor dynamic parasympathetic control may underlie certain DGBI (12). Several small studies have documented suboptimal autonomic regulation in both children and adult CVS patients (3, 13–15). A recent study demonstrated that children with CVS have abnormal cardiac vagal regulation during their inter-episodic well phase as compared to healthy controls (16).

There are no prior studies assessing the effects of PENFS on abdominal pain and extraintestinal comorbidities in CVS. By targeting autonomic pathways via stimulation of the outer ear, auricular neurostimulation may regulate triggers and comorbidities driven by altered sympathovagal balance. This study aimed to investigate the efficacy of PENFS on pain, anxiety, sleep, and quality of life in children with CVS. We hypothesized that in children with CVS, PENFS has positive effects on pain and comorbidities and, consequently, also improves quality of life.

## Methods

### Study population

This was a prospective, open-label trial in children aged 8–18 years with CVS followed up in an outpatient tertiary care CVS Center at Children's Wisconsin (CW) hospital between January 2018 and December 2020. All subjects met Rome IV and North American Society for Pediatric Gastroenterology, Hepatology and Nutrition (NASPGHAN) 2008 CVS criteria (1, 17). The study was approved by the CW Institutional Review Board. Informed consent/assent was obtained from all subjects and their legal guardian. All study interventions were performed in the CW pediatric Translational Research Unit.

All subjects underwent screening diagnostic tests as per the treating pediatric gastroenterologist. These tests were documented to be normal prior to CVS diagnosis per current expert consensus recommendations (1). Additional inclusion criteria were a CVS episode frequency of minimum every 6 weeks for at least two cycles prior to study enrollment and English language proficiency. Exclusion criteria were significant developmental delay, rumination syndrome, gastrointestinal motility disorder, seizure disorder, or other organic diseases that may elicit nausea and vomiting. No changes to patients’ existing pharmacologic therapy were allowed for at least 2 weeks prior to study enrollment and during the study period.

### Treatment protocol

A total of 32 subjects were enrolled. A PENFS device (Neuraxis, Carmel, IN, United States) was placed on the outer ear by a trained and certified research nurse as previously described (9). The PENFS device consists of a battery-operated unit connected to a harness with four titanium electrodes that are applied percutaneously to the external ear. The settings deliver 3.2 V in a rectangular pulse wave and alternating frequencies (1 ms pulses of 1 and 10 Hz) every 2 s. PENFS therapy was initiated during the baseline inter-episodic wellness period. Each device was worn for 5 days (24 h a day) for 6 consecutive weeks and removed by the family in the home setting. The frequency of CVS episodes was assessed at baseline and at an extended follow-up visit 4–6 months after end of the study. Subjects completed the following patient-reported questionnaires at various time points before and after the treatment intervention:

1. Abdominal Pain Index (18) (API): four items assessing frequency, duration, and intensity of abdominal pain over the past week, with score range 0–4 (higher score indicating greater symptom burden). The API was completed at baseline, weekly during therapy, at the end of therapy (week 6), and at extended follow-up.
2. Daily worst and average abdominal pain intensity on a scale from 0 to 10. Weekly averages were computed during the 6-week therapy for both worst and average pain intensity.
3. State-Trait Anxiety Inventory for Children (19) (STAI-C). This validated 40-item instrument measures both the current state of anxiety and the anxiety trait of a child. A lower score indicates less anxiety. The STAI-C was completed at baseline, end of therapy (week 6), and at extended follow-up.
4. Pittsburgh Sleep Quality Index (20) (PSQI). The adolescent version of this nine-item questionnaire was used to assess quality and patterns of sleep over the past month. A lower score indicates improved sleep quality. The measure was completed at baseline, end of therapy (week 6), and at extended follow-up.
5. Patient Reported Outcome Measurement Information System (21) (PROMIS) Pediatric Profile-37. This quality of life outcome measure efficiently assesses physical, emotional, and psychosocial functioning across six domains (Physical Function, Anxiety, Depression, Fatigue, Peer Relationships, and Pain Interference). A lower score indicates improved quality of life for the Anxiety, Depression, Fatigue, and Pain Interference subscales while a higher score relates to improvement in the Physical Function and Peer Relationships subscales. The measure was completed at baseline, weekly during the study, end of therapy (week 6), and at extended follow-up. They survey assessed these outcomes in relation to a typical CVS episode in order to better capture disease-specific quality of life.

### Statistical analysis

Continuous variables are summarized as median and interquartile range (IQR), and categorical variables as number (%). The Wilcoxon rank test was used to examine the score changes from time to time. A general linear model of repeated measures was used to examine the weekly averages of daily pain score and weekly API score changes over the treatment weeks. The maximum likelihood estimation method was used, and the subjects were viewed as clusters with heterogeneous autoregressive used as the variance covariance matrix. Pearson correlations test was used to examine any relationship between episode frequency (converted to monthly value) and outcome measures. A result was reported as significant for an unadjusted two-sided *p*-value <0.05. All data analyses were performed using SAS9.4 and SPSS28.0.

## Results

Thirty-two subjects were enrolled. One subject dropped out after first visit due to transportation issues and one was excluded from analyses due to incorrect diagnosis (constipation). Thirty subjects were included in the final analyses. Eighteen (60%) were female and median (IQR) age was 10.5 (8.5–15.5) years. The majority (*n* = 29, 97%) were Caucasian and one subject was Hispanic. Twenty-two subjects (73%) suffered from CVS attacks 1–2 times per month, while the rest had episodes as often as three times (*n* = 1) or four times (*n* = 7) per month. The median (IQR) duration of episodes was 48.0 (24.0–96.0) h. All participants had failed previous pharmacological interventions to control the CVS episodes with a median (IQR) of 2.5 (1.0–4.0) prophylactic medications failed prior to entering the trial. The median (IQR) time to extended follow-up was 4.4 (3.1–6.8) months.

The frequency of episodes/month decreased from monthly median (IQR) 2.0 (1.0–3.0) at baseline to 0.5 (0.2–1.13) at the extended follow-up (*p* < 0.0001).

### Pain measures

#### Abdominal Pain Index

**Figure 1:**
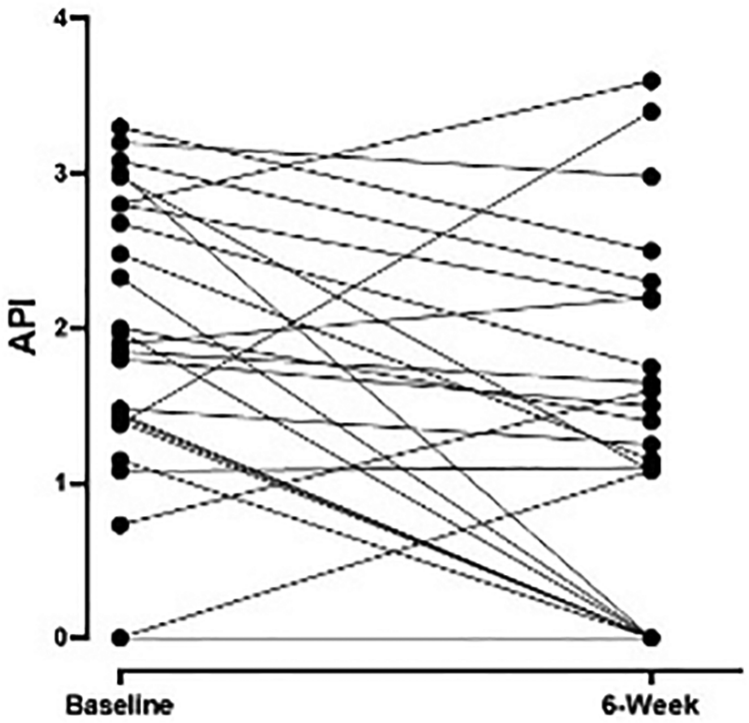
Weekly pain scores as measured by API during the treatment intervention with significant improvement after 6 weeks of therapy (*n* = 26 subjects) compared to baseline (*p* = 0.003). API, abdominal pain index.

Median (IQR) API scores on *n* = 26 subjects with complete data decreased from 1.64 (0–2.68) at baseline to 1.08 (0–1.65) at week 6 (*p* = 0.003) and to 0.55 (0–1.0) at follow-up (*p* < 0.0001; Figure 1). Weekly median API scores improved during the treatment course and were significantly lower than baseline scores after all treatment weeks (*p* < 0.05) except week 2 (*p* = 0.106).

#### Daily pain intensity

Weekly averages across the 6-week treatment intervention also showed a reduction in the daily mean abdominal pain scores from baseline median (IQR) 5.0 (1.0–6.0) to 0.36 (0–1.57) at week 6 (*p* < 0.0001). The baseline scores were higher than all subsequent weeks of therapy (*p* < 0.0001), and the weekly average for mean abdominal pain during week 1 was significantly higher than week 3 (*p* = 0.028). There were similar findings for weekly averages for daily worst abdominal pain with significantly higher score at baseline compared to all subsequent weeks (*p* < 0.0001) and higher scores at week 1 compared to week 3 (*p* = 0.011).

### State-Trait Anxiety Inventory for Children

**Figure 2:**
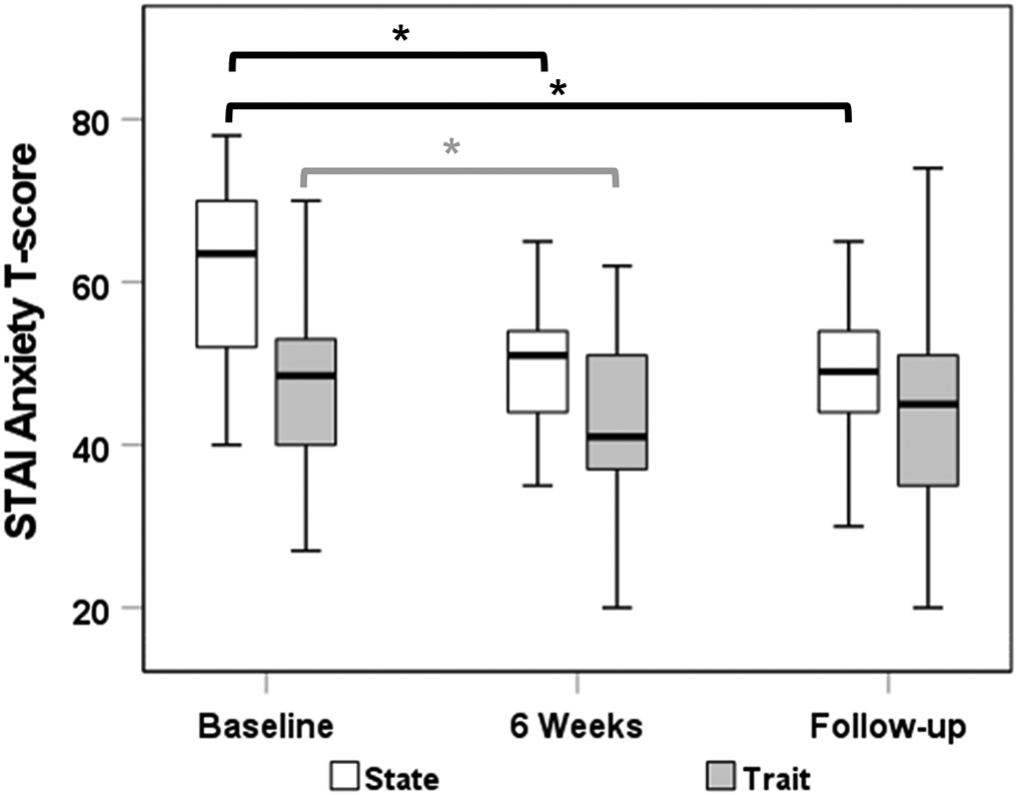
STAI-C anxiety state and trait *T*-scores from baseline to end of therapy and follow-up time point. STAI-C, State-Trait Anxiety Inventory for Children.

A total of 26 subjects had complete data for the STAI-C state measure at all time points. Median (IQR) state anxiety scores decreased from 63.5 (52.0–70.0) at baseline to 51.0 (44.0–54.0) at week 6 (*p* < 0.0001) and 49.0 (44.0–54.0) at follow-up (*p* < 0.0001). Similarly, median (IQR) trait anxiety scores (*n* = 25 with complete data) decreased significantly from 49.0 (44.0–53.0) at baseline to 41.0 (37.0–51.0) at week 6 (*p* = 0.031). There was no significant reduction in trait anxiety score from baseline to follow-up (*p* = 0.06; Figure 2). While there was no significant relationship between reduced STAI-C state scores and episode frequency, the decrease in STAI-C trait at follow-up correlated with decrease in the average episode frequency (*r* = 0.41; *p* = 0.041).

### Pittsburgh Sleep Quality Index

**Figure 3:**
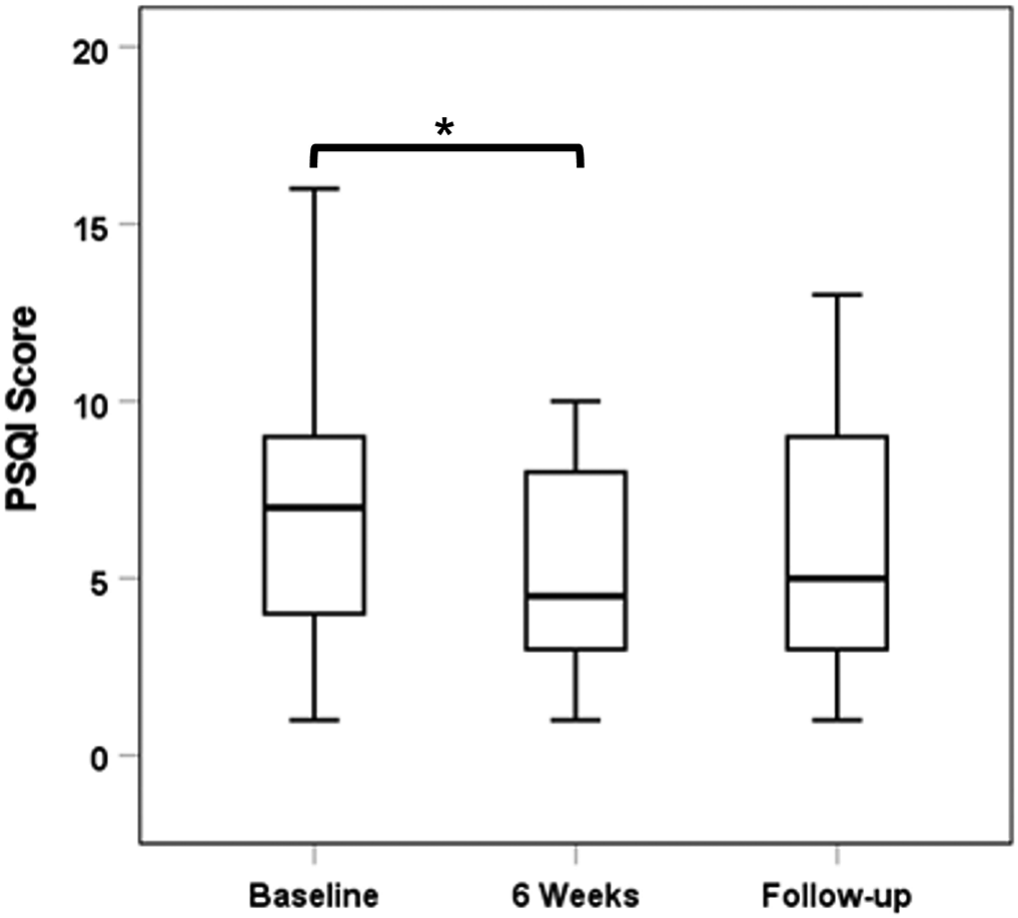
Median PSQI scores across study period. PSQI. Pittsburgh Sleep Quality Index.

Twenty-six out of the 30 subjects had complete data sets with regard to PSQI scores. Study participants showed improved sleep quality with PENFS treatment although this did not have long-term effects. The median (IQR) PSQI score decreased from 7.0 (4.0–9.0) at baseline to 4.5 (3.0–8.0) at 6 weeks (*p* = 0.031). When compared to baseline, there was a nonsignificant reduction in PSQI scores at extended follow-up: median (IQR): 5.0 (3.0–9.0) (*p* = 0.22; Figure 3).

### PROMIS Pediatric Profile-37

A total of 27 out of the 30 patients studied had complete data sets to analyze. Study participants showed improvement in disease-specific physical function, anxiety, fatigue, and pain interference after 6 weeks of PENFS therapy. PROMIS scores for the anxiety subscale were also significantly reduced at the follow-up time point compared to baseline (Table 1). There was a nonsignificant correlation between reduction in anxiety and improved episode frequency/month at the extended follow-up (Pearson *r* = 0.12; *p* = 0.54).

**Table 1:** PROMIS quality of life measure scores across several domains comparing baseline to 6 weeks and baseline to extended follow-up.

PROMIS subscales	Baseline median (IQR)	6 weeks median (IQR)	*p*-value*	Follow-up median (IQR)	*p*-value**
Physical function	25.4 (22.8–28.9)	27.8 (22.8–40.6)	**0** **.** **032**	27.8 (21.3–33.3)	0.064
Anxiety	57.4 (50.8–70.0)	44.7 (34.4–57.4)	**0** **.** **0002**	55.8 (39.2–62.1)	**0** **.** **046**
Depression	55.1 (50.0–61.9)	50.0 (45.3–59.3)	0.14	56.5 (42.4–63.2)	0.5
Fatigue	71.6 (58.8–80.7)	65.0 (32.8–73.5)	**0** **.** **003**	68.2 (57.3–80.7)	0.4
Peer relationships	46.7 (39.4–58.0)	46.7 (42.1–63.2)	0.54	46.7 (42.1–63.2)	0.47
Pain interference	67.2 (59.3–72.5)	61.8 (51.9–67.2)	**0** **.** **01**	64.4 (55.7–76.0)	0.64

PROMIS, Patient Reported Outcome Measurement Information System; IQR, interquartile range.

Bolded values denote statistical significance at *p* < 0.05.

* Baseline to 6 weeks comparisons.

** Baseline to follow-up comparisons.

Weekly median subscale scores improved significantly (*p* < 0.05) during the treatment course for physical function (baseline > all weeks), anxiety (baseline > all weeks), depression (baseline > weeks 1, 3, and 4), fatigue (baseline > weeks 1, 3, 5, and 6), and pain interference (baseline > all weeks).

## Discussion

This is the first study evaluating the effects of a non-implantable, auricular neurostimulation device on comorbidities and triggers associated with cyclic vomiting syndrome in children. This open-label, prospective trial demonstrates benefits of PENFS on pain, sleep, and several quality of life indices, including physical function, anxiety, fatigue, and pain interference. More importantly, there were long-term improvements in pain and anxiety measures at extended follow-up 4–6 months after study completion. The combination of short-term improvement in comorbidities and quality of life measures with a durable reduction in anxiety suggests that the therapy may have sustained alterations on the course of this disease.

Existing literature demonstrates a strong influence of mental health conditions in children with CVS. Anxiety is not only highly prevalent in children with CVS but also the main factor that predicts a reduced quality of life (5, 6). Anxiety and emotional arousal during positive and negative life events are the most common triggers for CVS attacks in children (3). A baseline higher autonomic reactivity and sensitivity to any disruptions of internal regulation is a postulated explanation for the rapid deterioration after an inciting trigger (22). Autonomic dysfunction has been implicated in the pathophysiology based on several smaller studies of both children and adults with CVS (3, 13–15). One small study documented reduced parasympathetic tone in response to a social stress test (3). This study further noted that majority (82%) of children with CVS met the criteria for anxiety and that acute (state) anxiety (but not trait anxiety) correlated negatively with parasympathetic tone. These findings support a link between acute stress responses and altered vagal reactivity in children with CVS. Recently published data demonstrate an underlying autonomic imbalance with inefficient cardioinhibitory vagal regulation in children with CVS during their interictal well phase (16). These findings were generated in comparison to a larger cohort of age-, gender-, and size-matched healthy controls and corrected for medication confounders. Although speculative, anxiety may thus induce an unimpeded sympathetic activation that is chronically dampened by PENFS therapy, which likely acts via stimulation of the auricular branch of the vagus nerve (12). Thus, the long-term effects on anxiety measures in this study corroborate prior data and a plausible therapeutic mechanism that targets the underlying autonomic dysregulation. PENFS may have dual mechanistic roles by (1) influencing autonomic nervous system (ANS) and thus targeting the underlying pathology of CVS (16) and secondarily reducing anxiety/worries about subsequent episodes, and/or (2) direct anti-anxiety effects via vagal neurostimulation as reported in both human and animal studies (23, 24) and thus minimizing the triggering events. It is interesting to note that CVS has been linked to altered brain network connectivity. In adults with CVS, a functional MRI study suggested increased connectivity between the salience processing network and the mid/posterior insula, a key region for processing nausea and viscerosensory stimuli (25). There was also decreased sensorimotor connectivity in both CVS and migraine patients, suggesting a common pathophysiology. This provides another potential mechanism of excessive neuronal excitability with a lower threshold for activation by triggering events. Vagal neuromodulation via PENFS may induce neural plasticity and alter brain connectivity (26), thereby dampening this hyperexcitability. In a study of adults with fibromyalgia undergoing PENFS therapy, improved pain interference and sleep correlated with altered resting state functional network connectivity (27).

There is a great demand for effective, non-pharmacological therapies for children with CVS. Recent data show that children with DGBI incur significant healthcare utilization due largely to the lack of targeted and effective therapies (28). CVS patients, in particular, incur a substantial healthcare burden due to repeated emesis attacks (29). Costs related to hospitalizations for children with acute episodes of CVS have more than doubled over the past two decades and totaled $84 million/year (30). The need for repeated high acuity medical interventions due to rapid dehydration is likely to escalate the mental health comorbidities, explaining why anxiety is the main contributor to poor quality of life and disability (6). While there are scant supportive data and no regulatory approval, children with CVS are frequently treated with antidepressants that incur the risks of serious side effects and polypharmacy (8, 31, 32). A Cochrane review found no evidence to support the use of antidepressants for children with DGBI (7). Further, these drugs have strong anticholinergic effects that exert direct, inhibitory effects on cardiac vagal signaling (33). Finding effective, nondrug alternatives is thus paramount to improving care for this disabling condition.

The strengths of our study include the prospective study design and the long-term and multidimensional assessment of disease comorbidities and quality of life indices. Yet, our study has several important limitations. The lack of a sham-controlled study design is an important limitation and placebo effects may underlie some of the improvement as subjects received weekly attention. Placebo effects may be particularly high with non-pharmacological, noninvasive interventions such as the PENFS that are often highly sought by parents of CVS sufferers after failing numerous drug therapies. Bias in the report of symptom improvement may thus be a factor. Yet, the device placements were performed by certified research nurses who are not part of the research team and were instructed to refrain from interactions with subjects. The noted long-term improvements are also less likely due to placebo effects. Assessing an episodic illness with standardized patient-reported outcome surveys designed for chronic conditions is problematic. The pain assessment (API), in particular, is a survey of pain symptoms over past week, and long-term improvement in this measure may be less relevant to an episodic illness. However, progressive pain improvement throughout and after the treatment intervention based on several measures coupled with long-term reduction in episode frequency suggests that PENFS therapy influences the disease course. Additionally, the inclusion of comprehensive and long-term assessments of several comorbidities and quality of life measures in relation to the CVS episodes lessens this confounder. State anxiety assessment could also be confounded by the assessment at single points in time. The state anxiety or PROMIS anxiety subscales did not show a clear relationship. This could be due to a small sample size and insufficiently powered study. A longer duration of follow-up is likely needed to thoroughly assess the impact on episode frequency. However, anxiety indices were improved on both PROMIS and STAI-C (state) scales, suggesting long-term improvement in this common comorbidity. Further investigations of the impact of mental health comorbidities, including antecedent trauma, would be important for a more comprehensive understanding of this patient cohort. Finally, that lack of standardization of protocols, the type of stimulation (transcutaneous vs. percutaneous), and stimulation parameters limits the generalizability of research in the field of noninvasive neuromodulation (34). Reliable biomarkers of parasympathetic activity are needed to optimize stimulation protocols and individualize therapy.

In summary, this pilot study shows promising effects of noninvasive auricular neurostimulation via PENFS in children with CVS. While short-term effects were noted for several pain and quality of life measures during and after therapy, there were important long-term improvements on several anxiety measures. As a sympathetically mediated disorder strongly governed by heightened arousal and stress states, this therapy shows promise as an emerging non-pharmacological and targeted intervention for CVS.

## Data Availability

The original contributions presented in the study are included in the article, further inquiries can be directed to the corresponding author.
